# Bread, wholegrain consumption and weight change from middle to late adulthood: a prospective cohort study

**DOI:** 10.1007/s00394-025-03724-8

**Published:** 2025-05-30

**Authors:** Ingrid Revheim, Zoya Sabir, Jutta Dierkes, Anette E. Buyken, Rikard Landberg, Inka Alten, Ulrike Spielau, Hanne Rosendahl-Riise

**Affiliations:** 1 Centre for Nutrition, Department of Clinical Medicine, University of Bergen, Post box 7804, Bergen, 5020 Norway; 2 Institute of Nutrition, Consumption and Health, Faculty of Natural Sciences, Paderborn University, Paderborn, Germany; 3 Division of Food and Nutrition Science, Department of Life Sciences, Chalmers University of Technology, Gothenburg, Sweden

**Keywords:** Bread, Whole grains, Alkylresorcinols, Weight change, Epidemiology

## Abstract

**Purpose:**

Bread is a global staple with large variations in carbohydrate quality. The role of bread for weight regulation has been controversial and few studies have investigated associations of bread consumption with long-term weight changes. Therefore, our objective was to investigate the associations of baseline intake of bread, different types of bread, and whole grains as well as plasma alkylresorcinol – a biomarker of wholegrain wheat and rye intake – with weight changes during a follow-up covering the 6th and 7th decades of life.

**Methods:**

The current analyses were conducted in 1764 men and women (47.4 ± 0.6 years at baseline) participating in the second and third wave of the Hordaland Health Study. A food frequency questionnaire was used to estimate baseline bread and wholegrain intake. Baseline plasma alkylresorcinol concentrations were determined in blood samples. The outcome was weight change (kg) during the 20-year follow-up. Multivariate linear regression models were applied.

**Results:**

Participants gained on average 2.1 ± 7.4 kg during follow-up. While total bread intake at baseline was not associated with weight change, a higher white bread intake was associated with weight gain (0.017 kg/g white bread/day, 95% CI: 0.002, 0.032 kg/g white bread/day). Wholegrain consumption – but not wholegrain bread – was inversely associated with weight gain (-0.013 kg/g whole grain/day, 95% CI: -0.026, 0.000 kg/g whole grain/day). Accordingly, plasma alkylresorcinols were inversely associated with weight gain (-0.004 kg/nmol/L alkylresorcinol, 95% CI: -0.007, -0.002 kg/nmol/L alkylresorcinol).

**Conclusion:**

The current study suggests that weight regulation is associated with the carbohydrate quality of breads, i.e., refined versus wholegrain breads. Wholegrain intake, based on both self-reported dietary data and objective biomarkers, may improve weight regulation from middle to late adulthood.

**Supplementary Information:**

The online version contains supplementary material available at 10.1007/s00394-025-03724-8.

## Introduction

Bread is a major staple worldwide and one of the main sources of whole grains and dietary fibre in many countries. However, the carbohydrate quality of bread largely varies depending on the degree of refinement and the type of grain used. A high consumption of white bread has been associated with weight gain [1], and consuming two portions of white bread daily, compared to one or less, has been associated with a 40% higher risk of developing overweight or obesity [2]. Contrary, higher wholegrain intakes have been associated with a range of beneficial health outcomes in observational studies [3–5], including lower body weight [6], lower body mass index (BMI) [7, 8], and lower weight gain over time [9–11]. Furthermore, wholegrain bread consumption has not been associated with weight gain [1] nor with the risk of developing overweight or obesity [2] in prospective cohort studies. These findings highlighting the need for detailed analyses of the carbohydrate quality, e.g., wholegrain and dietary fibre content, degree of refinement, and glycaemic index, of the bread consumed in relation to weight regulation.

The food frequency questionnaire (FFQ) is a common dietary assessment method applied in larger-scale epidemiological studies due to its affordability and feasibility. The FFQ reflects habitual dietary intake over a longer time period, and it allows for the estimation of energy and nutrient intake [12]. However, dietary intake is inherently difficult to measure and estimations of cereal foods and food components such as bread and whole grains based on self-reported methods may be prone to systematic and random measurement errors [12, 13]. Biomarkers are compounds derived from specific foods or food components serving as indicators of their consumption. When used in parallel with self-reported methods, biomarkers can provide complementary information as the two methods have uncorrelated measurement errors [14, 15]. Several biomarkers of different cereals and whole grains thereof have been suggested [16]. Alkylresorcinols (ARs), phenolic lipids derived from the bran of wheat and rye, show promising results as biomarkers for wholegrain wheat and rye intake [17–19]. ARs are not affected by food processing [20], they are absorbed in the small intestine [19], and they can be measured in plasma [21]. In addition to being used as biomarkers of compliance in intervention trials, plasma ARs have been suggested as useful biomarkers for assessing medium-to-long-term intake of wholegrain wheat and rye, two main cereals used in bread, in free-living populations [22, 23].

Weight changes across the lifespan have been associated with adverse health outcomes including accelerated biological aging [24], cardiometabolic risk factors [25], and mortality [26, 27]. Stable obesity across adulthood, weight gain from early to middle adulthood, and weight loss from middle to late adulthood have been associated with increased risk of mortality [26]. Thus, avoiding early onset obesity, reducing the obesity exposure across the lifespan, but also avoiding excessive weight loss in later adulthood may reduce the risk of adverse health outcomes.

Given the controversial role of bread consumption for weight regulation and the scarcity of scientific studies investigating the association of bread consumption with long-term weight changes, especially those combining quantitative dietary assessment methods with dietary biomarkers, the main objective of the current study was to investigate the association of bread intake at baseline with long-term weight change. This was achieved by following a cohort of Norwegian men and women from middle to late adulthood, covering the 6th and 7th decades of life. Secondary objectives were to investigate the associations of bread quality, total wholegrain intake, and total plasma AR concentration with long-term weight change.

## Methods

### Study population

**Fig. 1:**
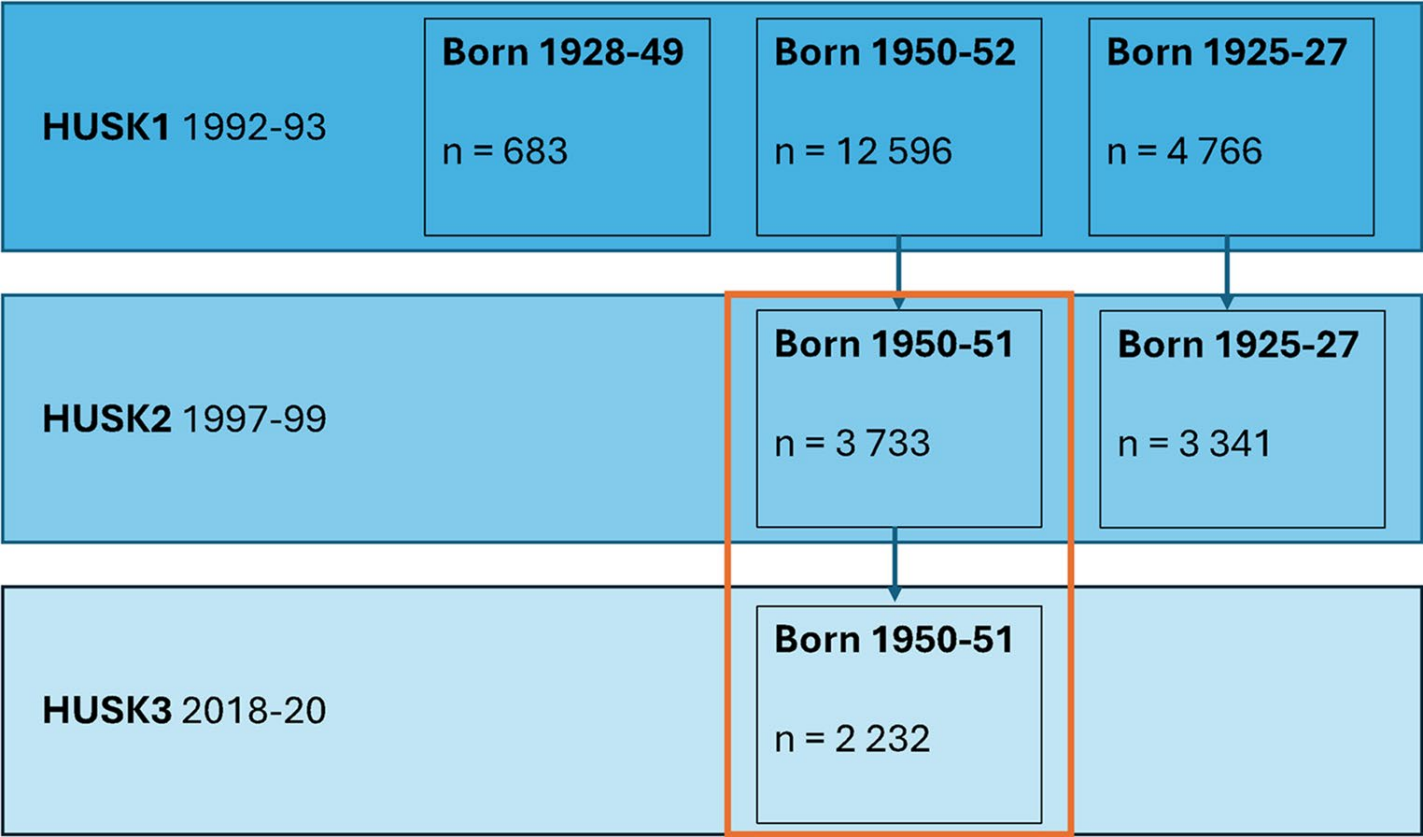
Flowchart showing the birth cohorts and number of participants attending the three study waves of the Hordaland Health Study. The orange box indicates that subjects born in 1950-51 participating in both HUSK2 and HUSK 3 were included in the statistical analyses

The Hordaland Health Studies are community-based health surveys conducted in Hordaland County, Western Norway. All residents of Hordaland County born in 1925-27 and 1950-52 were invited to participate in the Hordaland Homocysteine Study in 1992-93. In 1997-99, the two age cohorts were reinvited to participate in the Hordaland Health Study (HUSK2). In 2018-20, those born in 1950-51 were reinvited to take part in a follow-up health survey, HUSK3 (Fig. 1). Additional information may be found at https://husk.w.uib.no. In the current study, data from the 1950-51 cohort in HUSK2 and HUSK3 were used to explore the associations of bread intake, wholegrain intake, and plasma AR concentrations with weight change between HUSK2 (baseline) and HUSK3 (follow-up).

In total, 2232 participants born in 1950-51 attended both HUSK2 and HUSK3. Participants who completed the food frequency questionnaire (FFQ) and provided measurements of body weight and plasma AR in HUSK2, and who provided data on body weight in HUSK3, were included (*n* = 1799). Participants who reported a very low energy intake (< 3000 kJ/day for women and < 3300 kJ/day for men) or a very high energy intake (> 15 000 kJ/day for women and > 17 500 kJ/day for men), and participants with implausible total plasma AR values (> 1200 nmol/L), were excluded. After removal of participants with missing exposure and outcome data (*n* = 433), implausible energy intakes (*n* = 32), and implausible plasma AR values (*n* = 3), 1764 subjects were included in the statistical analyses.

### Dietary assessment

In HUSK2, habitual dietary intake was assessed using a paper-based self-administered, 169-item semi-quantitative FFQ developed by the Department of Nutrition, University of Oslo, Norway. The FFQ was designed to obtain information on the usual food intake during the past year. Adequate validity has been demonstrated for the FFQ regarding energy intake and most nutrients (median correlation coefficient of 0.6 between FFQ and weighted food records, with a respective coefficient of 0.5 for total carbohydrate intake) [28]. The frequency responses ranged from never to multiple times per day, week, or month. Portion sizes were estimated using household measures or units such as slices or cups, depending on the food item inquired. The daily intakes of foods, energy, and nutrients in grams per day (g/day) were estimated using the software system KostBeregningsSystemet (KBS, version 3.2, database IE-96, Department of Nutrition; University of Oslo, Norway) and its associated food composition table, which is an extended version of the Norwegian Food Composition Table [29].

### Bread intake

Bread intake was reported in slices per day, and based on the reported intake, the amount of bread eaten in g/day was estimated. The FFQ inquired about consumption of wholegrain bread, medium dark bread, refined white bread, and other types of bread (e.g., crispbread and flatbread). Generic bread types in the Norwegian Food Composition Table were used to reflect the intake of the different types of bread. Total bread intake was defined as the sum of the intake of wholegrain, medium dark, white, and other types of bread.

### Wholegrain intake

Using the Norwegian Food Composition Table, wholegrain intake was estimated by determining the wholegrain content of all grain foods based on the dry weight of whole grains in the specific food item inquired. The average daily wholegrain intake was calculated by multiplying the amount of whole grain in the respective food item in question by the reported daily intake. The main food items in the FFQ related to wholegrain intake included bread (wholegrain, medium dark, white, and crispbread) and cereal products (oats, cereals, porridge, and muesli). Of note, the FFQ did not distinguish between the degree of processing of the grain, and thus, the term “whole grain” as used in this paper covers both consumption of intact kernels (whole grains) and products made of milled flour (wholemeal).

### Clinical measures

The collection of clinical data was performed according to standard procedures. Height was measured without shoes in light clothing to the nearest cm using a stadiometer. A flexible measuring tape was used to measure waist circumference while participants stood upright, placed their feed together, and exhaled. Waist circumference was measured twice to the nearest 0.1 cm, and the mean was reported. Blood pressure was measured in a seated position after at least 5 min of rest. Three measurements were performed at one-minute intervals, and the mean of the two last readings was reported. Baseline blood pressure measurements were performed with a calibrated blood sphygmomanometer (Dinamap 845 XT Criticon, Tampa, USA). In both study waves, body weight was measured in light clothing without shoes on a calibrated digital scale. Measurements were obtained in a non-fasting state throughout the day, from early morning until the afternoon. Absolute weight change (kg) during follow-up was estimated based on the difference between body weight measured in HUSK2 and HUSK3. Stable weight was defined as < 5% change, whereas weight gain or loss was defined as ± ≥ 5% change, respectively.

### Biochemical measures

Venous blood samples at baseline were collected in a non-fasting state throughout the day (starting in the morning and ending in the afternoon). Plasma blood samples were collected in EDTA-tubes and stored at 4–5 °C until centrifuged, within one hour, at 4000 x g for ten minutes. The samples were stored at -80 °C until further analysis. Serum blood samples were collected in tubes containing sodium sulphite titration gel. The samples were centrifuged at 1000 x g for ten minutes within one hour and stored at -80 °C until further analysis. Analysis of serum glucose, total cholesterol, HDL-cholesterol, and triglycerides were performed at the Department of Clinical Chemistry, Oslo University Hospital, using enzymatic methods (Hitachi 911 chemistry analyser). The Friedewald equation was used to calculate LDL-cholesterol [30].

### Plasma alkylresorcinol concentrations

The concentrations of plasma AR homologues (C17:0, C19:0, C21:0, C23:0, C25:0) and their sum were determined by liquid chromatography – tandem mass spectrometry at the Division of Food and Nutrition Science, Department of Life Sciences, Chalmers University of Technology, Gothenburg, Sweden using the conditions reported by Ross et al. [31]. Quality control samples were included in each batch to ensure appropriate within- and between-batch precision. A coefficient of variation of < 10% was set as an acceptance criterion for the system suitability test.

### Lifestyle and sociodemographic factors

Self-administered questionnaires were used to collect information on current health, medical conditions, physical activity level, smoking status, marital status, and educational level. Changes in physical activity level during follow-up were obtained by comparing the overall physical activity level reported in HUSK2 with that reported in HUSK3. In both study waves, participants were asked to report the average hours/week (h/wk) of light intensity and vigorous intensity physical activity during the past year. Light intensity physical activity was weighted as “0” (0 h/wk), “0.25” (1 h/wk), “0.5” (2 h/wk), “0.75” (3 h/wk), “1” (≥ 4 h/wk). Vigorous intensity physical activity was weighted as “0” (0 h/wk), “0.5” (1 h/wk), “1” (2 h/wk), “1.5” (3 h/wk), and “2” (≥ 4 h/wk). The sum of light and vigorous intensity activity was used as an overall score of the physical activity level. Physical activity levels during follow-up were categorised into “less” (score in HUSK2 > score in HUSK3), “stable” (equal score in HUSK2 and HUSK3), and “more” (score in HUSK2 < score in HUSK3).

Current smoking in HUSK2 was defined as plasma cotinine concentrations ≥ 85 nmol/L [32] or self-reported smoking status for participants with missing cotinine values (*n* = 10). Changes in smoking habits during follow-up were categorised based on smoking status in HUSK2 and self-reported smoking status in HUSK3. Categories were “no-no” for those who never smoked, “yes-yes” for those smoking in both study waves, “no-yes” for those who started smoking, and “yes-no” for those who quitted during follow-up. Educational level was categorised into ≤ 12 years or > 12 years of education.

### Statistics

Normality was assessed for all continuous variables by visual inspection of histograms and Q-Q plots. Participant characteristics are described using summary statistics; continuous variables are expressed as mean and standard deviations, and categorical variables as counts and percentages. Highly skewed continuous variables are expressed as medians with the corresponding 25th and 75th percentiles. ANOVA and chi-square tests were used to compare participant characteristics across quartiles of sex-specific bread intake for continuous and categorical variables, respectively. For non-normally distributed variables, the Kruskal-Wallis H test was used for comparison.

The outcome assessed was change in body weight (kg) over a period of 20 years. Multivariate linear regression models were applied to examine the association between (1) total bread intake (g/day), (2) wholegrain bread intake (g/day), (3) white bread intake (g/day), (4) wholegrain intake (g/day), and (5) total plasma AR concentrations (nmol/L) at baseline with changes in body weight (kg) during follow-up. The relationships between the exposure variables and the outcome variable were assessed by visual inspection of scatterplots and by curve estimation regression models. The variables appeared to be linearly related, and thus, a linear regression model was chosen. The exposure variables were expressed as continuous variables (g/day), and for total bread and wholegrain intake, both continuous and categorical (sex-specific quartiles) variables were applied. Sex-specific quartiles were used to get an equal distribution of males and females across the quartiles. The outcome variable, weight change (kg) during follow-up, was used as a continuous variable.

In the regression analyses, Model 1 was adjusted for baseline body weight, Model 2 was adjusted for baseline body weight, sex, and partial energy intake at baseline, and Model 3 was adjusted for body weight, sex, partial energy intake, and education obtained at baseline, and changes in smoking habits and physical activity levels during follow-up. Changes in both smoking habits and physical activity levels can be viewed as predictors of weight change as they may influence weight directly or interact with other variables to affect weight [33, 34]. Thus, changes in smoking habits and level of physical activity during follow-up, rather than baseline levels, were included as risk factors for weight change in the regression models. Energy intake was adjusted using the partition model, which allows for the estimation of the effect of the exposure of interest whole keeping the remaining energy intake constant. This approach is commonly used in nutritional epidemiology when investigating specific food components while accounting for total energy intake [35, 36]. The regression models with plasma AR as exposure were adjusted for total energy intake. Additionally, these models were also adjusted for triglyceride levels (mmol/L) measured at baseline as triglycerides serve as a predictor variable for plasma ARs. The regression models including sex-specific quartiles of exposure were not adjusted for sex.

Missing data (*n* = 2 for smoking, *n* = 11 for education, and *n* = 84 for physical activity level) were accounted for by applying imputation methods where the sex-specific median and mode values for continuous and categorical variables, respectfully, were used to replace missing data.

As the overall baseline intake of white bread was low, sensitivity analyses including only those who reported consuming white bread in the past year (*n* = 533) were conducted. Furthermore, as the main analyses incorporated changes in smoking habits and physical activity levels during follow-up as covariates, sensitivity analyses using smoking status and physical activity level at baseline were conducted. Sensitivity analyses were also conducted where those who started smoking during follow-up (*n* = 6) were excluded from the statistical analyses as the small size of this group could introduce some instability in the statistical model. The “new smokers” were excluded rather than combined with another group as smoking habits have a large impact on body weight, and thus, combining categories might introduce bias. These analyses can be viewed in Online Resource 1.

Spearman’s rank correlation was used to assess the relationship between wholegrain intake (g/day) and total plasma AR values (nmol/L) in HUSK2, as well as the relationship between bread and wholegrain intake at baseline (HUSK2) and follow-up (HUSK3).

Statistical analyses were conducted in IBM^®^ SPSS Statistics for Windows, version 29 (Armonk, NY: IBM Corp.). All tests were two-tailed with a significance level of 0.05.

## Results

### Participant characteristics

The study included 1764 participants of whom 1024 (58%) women and 740 (42%) men. The median duration of follow-up was 20 years, ranging from 19 to 22 years. The baseline consumption of bread was high in the study cohort, and all but nine participants reported eating at least one slice of bread (~ 40 g) daily. Mean bread intake at baseline corresponded to 4–5 slices of bread daily. Wholegrain bread and medium dark bread contributed to 87% of the total bread intake. Participant characteristics by quartiles of bread intake are shown in Table 1, whereas descriptive nutritional intakes at baseline are shown in Table 2.

**Table 1:** Descriptive characteristics by sex-specific quartiles of total bread intake, the Hordaland health studies

Total bread intake (g/day)	Total cohort (*n* = 1764) 185 ± 76	Q1 (*n* = 442) 108 ± 32	Q2 (*n* = 442) 158 ± 28	Q3 (*n* = 440) 201 ± 37	Q4 (*n* = 440) 274 ± 69	*p* for trend
Women, *n* (%)	1024 (58.0)	257 (58.1)	257 (58.1)	255 (58.0)	255 (58.0)	> 0.99
Age, years	47.4 ± 0.6	47.4 ± 0.6	47.4 ± 0.6	47.4 ± 0.6	47.4 ± 0.6	0.685
Height, cm	171.6 ± 9.0	170.8 ± 9.0	171.0 ± 0.9	171.8 ± 8.9	172.7 ± 9.0	**0.008**
Weight, kg	74.6 ± 13.6	75.6 ± 14.2	74.3 ± 13.8	74.4 ± 13.2	74.1 ± 13.0	0.369
BMI, kg/m^2^	25.2 ± 3.6	25.8 ± 3.7	25.3 ± 3.5	25.1 ± 3.5	24.8 ± 3.5	**< 0.001**
Waist circumference, cm	85.1 ± 11.2	86.1 ± 11.9	84.7 ± 11.1	84.9 ± 11.1	84.6 ± 10.7	0.141
Weight change, kg	2.1 ± 7.4	2.5 ± 7.3	2.0 ± 7.3	1.7 ± 7.1	2.3 ± 7.9	0.355
*Stable ± < 5%*,* n* (%)	767 (43.5)	185 (41.9)	201 (45.5)	197 (44.8)	184 (41.8)	0.576
*Loss ≥ 5%*,* n* (%)	343 (19.4)	82 (18.6)	83 (18.8)	91 (20.7)	87 (19.8)	0.847
*Gained ≥ 5%*,* n* (%)	654 (37.1)	175 (39.6)	158 (35.7)	152 (34.5)	169 (38.4)	0.380
Systolic blood pressure, mmHg	126.2 ± 14.7	126.8 ± 14.7	125.3 ± 13.7	125.9 ± 14.5	126.7 ± 16.0	0.406
Diastolic blood pressure, mmHg	74.0 ± 10.5	74.8 ± 10.8	73.5 ± 9.5	73.7 ± 10.6	74.2 ± 11.2	0.265
Serum blood glucose, mmol/L	5.2 ± 0.9	5.1 ± 0.9	5.1 ± 0.8	5.2 ± 1.0	5.2 ± 1.0	0.538
Total serum cholesterol, mmol/L	5.7 ± 0.9	5.8 ± 0.9	5.7 ± 1.0	5.6 ± 0.9	5.6 ± 1.0	**0.036**
HDL-cholesterol, mmol/L	1.3 ± 0.4	1.4 ± 0.4	1.3 ± 0.4	1.4 ± 0.4	1.3 ± 0.4	0.448
LDL-cholesterol, mmol/L	3.6 ± 0.9	3.7 ± 0.9	3.7 ± 0.9	3.5 ± 0.8	3.5 ± 0.9	**0.019**
Triglycerides, mmol/L	1.4 (1.0, 2.0)	1.4 (1.0, 2.0)	1.4 (1.0, 1.9)	1.3 (1.0, 1.9)	1.4 (0.9, 2.0)	0.992
Smoking						
*Current smoking at baseline*,* n* (%)	531 (30.1)	142 (32.1)	121 (27.4)	133 (30.2)	135 (30.7)	0.476
Smoking habits during follow-up						
*Never smoked*,* n* (%)	1218 (69.1)	296 (67.0)	317 (71.7)	305 (69.3)	300 (68.2)	0.418
*Continued smoking*,* n* (%)	114 (6.5)	33 (7.5)	21 (4.8)	29 (6.6)	31 (7.1)	0.371
*Quitted smoking*,* n* (%)	424 (24.0)	111 (25.1)	102 (23.1)	104 (23.6)	107 (24.3)	0.880
*Started smoking*,* n* (%)	6 (0.3)	2 (0.5)	1 (0.2)	1 (0.2)	2 (0.5)	0.905
Education > 12 years, *n* (%)	807 (45.7)	201 (45.5)	217 (49.1)	196 (43.9)	193 (43.9)	0.408
Physical activity during follow-up						
*Stable*,* n* (%)	394 (22.3)	101 (22.9)	91 (20.6)	109 (24.8)	93 (21.1)	0.437
*Decreased*,* n* (%)	383 (22.8)	112 (25.3)	90 (20.4)	88 (20.0)	112 (25.5)	0.077
*Increased*,* n* (%)	917 (54.6)	229 (51.8)	261 (59.0)	243 (55.2)	235 (53.4)	0.158
Marital status						
*Married*	1380 (78.2)	345 (78.1)	339 (76.7)	354 (80.5)	342 (77.7)	0.580

Descriptive characteristics are obtained in the second wave of the Hordaland Health Study (HUSK2), except for weight change, categories of weight change, change in smoking habits, and change in physical activity level which were also based on data collected in the third study wave (HUSK3)

Continuous variables are presented as mean ± standard deviation. Highly skewed continuous variables are presented as median with corresponding 25 and 75 percentiles. Categorical variables are presented as counts with percentages

AR, alkylresorcinol; BMI, body mass index; HDL, high-density lipoproteins; LDL, low-density lipoproteins

^a^*P*-values for continuous variables are obtained from ANOVA for normally distributed data and from Kruskal-Wallis test for highly skewed data. Categorical variables are compared by the chi-square test

Several distinctions were present when comparing participants across quartiles of total bread intake including differences in height, BMI, total cholesterol, and triglycerides (Table 1). Changes in smoking habits and physical activity levels during follow-up did not differ across quartiles, nor did changes in body weight. Following the higher bread intake, intakes of whole grains and dietary fibre, and total plasma AR concentrations, were higher in the higher quartiles of bread intake compared with the lower ones. Overall, intake of food, macronutrients, and energy was higher in the higher quartiles of bread consumption, except for alcohol consumption which was lower (Table 2).

**Table 2:** Baseline food, nutrient, and energy intakes estimated by a food frequency questionnaire and reported by sex-specific quartiles of total bread intake, the Hordaland health studies

Total bread intake, g/day	Total cohort (*n* = 1764) 185 ± 76	Q1 (*n* = 442) 108 ± 32	Q2 (*n* = 442) 158 ± 28	Q3 (*n* = 440) 201 ± 37	Q4 (*n* = 440) 274 ± 69	*p* for trend^a^
Energy, kJ/day	8955 ± 2543	7640 ± 2222	8471 ± 2045	9156 ± 2332	10,564 ± 2603	**< 0.001**
Wholegrain bread, g/day	115 ± 94	49 ± 47	79 ± 64	128 ± 74	204 ± 99	**< 0.001**
Medium dark bread, g/day	51 ± 68	39 ± 46	59 ± 62	55 ± 75	49 ± 81	**< 0.001**
White bread, g/day	0 (0, 13)	0 (0, 13)	0 (0, 25)	0 (0, 13)	0 (0, 13)	0.520
Other breads, g/day	5 (1, 13)	4 (0, 15)	5 (1, 12)	5 (1, 13)	5 (1, 13)	0.566
Wholegrain, g/day	58 ± 28	45 ± 28	50 ± 22	62 ± 25	76 ± 25	**< 0.001**
Total plasma AR, nmol/L	98.0 (52.8, 188.6)	81.9 (39.6, 153.2)	87.4 (46.8, 173.1)	106.0 (59.8, 205.7)	118.9 (71.6, 213.6)	**< 0.001**
Carbohydrates, g/day	262 ± 76	216 ± 63	246 ± 62	269 ± 65	316 ± 77	**< 0.001**
Dietary fibres, g/day	24 (19, 29)	20 (16, 26)	22 (18, 27)	25 (21, 29)	28 (24, 34)	**< 0.001**
Sugar, g/day	32 (18, 50)	33 ± 27	37 ± 29	36 ± 24	42 ± 28	**< 0.001**
Protein, g/day	84 ± 24	74 ± 23	80 ± 21	86 ± 23	96 ± 25	**< 0.001**
Fat, g/day	78 ± 28	67 ± 25	74 ± 23	79 ± 27	92 ± 30	**< 0.001**
Saturated fat, g/day	30 ± 11	26 ± 10	29 ± 9	31 ± 11	35 ± 12	**< 0.001**
Monounsaturated fat, g/day	25 ± 9	22 ± 8	24 ± 8	25 ± 9	29 ± 9	**< 0.001**
Polyunsaturated fat, g/day	17 ± 8	14 ± 6	16 ± 7	17 ± 7	21 ± 9	**< 0.001**
Vegetables, g/day	177 (116, 278)	180 (117, 291)	175 (113, 282)	181 (119, 275)	174 (113, 268)	0.536
Fruit and berries, g/day	220 (135, 330)	197 (117, 291)	223 (135, 341)	228 (156, 313)	230 (141, 357)	0.003
Fish, g/day	71 (47, 100)	64 (43, 97)	69 (46, 96)	70 (49, 102)	78 (51, 110)	**< 0.001**
Meat, g/day	121 ± 58	112 ± 56	119 ± 52	127 ± 62	127 ± 60	**< 0.001**
Alcohol, g/day	4.0 (1.1, 9.0)	4.8 (1.8, 9.6)	4.7 (1.4, 9.0)	3.6 (1.4, 9.0)	3.0 (0.4, 7.8)	**< 0.001**

Information on food and nutrient intakes were obtained in the second wave of the Hordaland Health Study. Variables are presented as mean ± standard deviation. Highly skewed variables are presented as median with corresponding 25 and 75 percentiles

^a^*P*-values were obtained from ANOVA, and for highly skewed data, from the Kruskal-Wallis H test

### Weight change

The average weight gain was 2.1 ± 7.4 kg during follow-up. Women had a mean weight gain of 2.5 ± 7.3 kg, whereas men had a weight gain of 1.6 ± 7.5 kg. 39% of the women and 49% of the men had a stable weight during follow-up. 19% of the women and 20% of the men lost ≥ 5% of initial body weight, whereas 42% and 31% of the women and men, respectively, gained ≥ 5% of initial body weight during follow-up.

### Bread intake and weight change

Total bread, wholegrain bread, and white bread consumption at baseline was significantly and positively correlated with total bread, wholegrain bread, and white bread consumption at follow-up (Table 3).

**Table 3:**
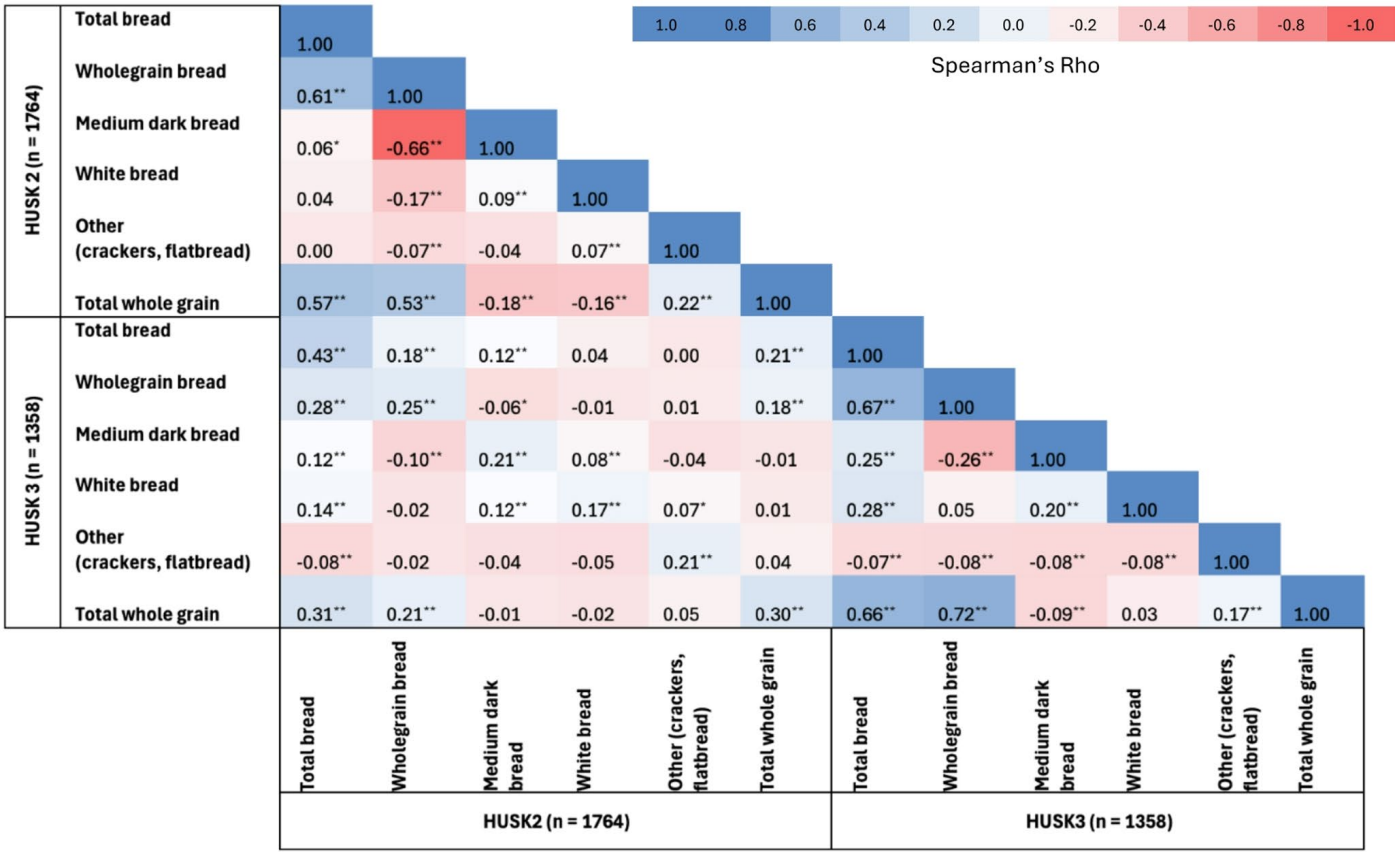
Correlations between bread and wholegrain intake at baseline and follow-up in Norwegian men and women participating in the Hordaland health studies

Correlation coefficients and *p*-values were estimated using Spearman’s rank-order correlation as the two food frequency questionnaires (FFQ) used at baseline and follow-up differed in number of food items inquired. Dietary intake data at baseline (HUSK2) was obtained by a paper-based 169-item FFQ. Dietary intake data at follow-up (HUSK3) was obtained by a 279-item web-based FFQ. Dietary data was available for 1764 subjects in HUSK2 and 1358 subjects in HUSK3. ** Correlation is significant at the 0.01 level (2-tailed). *Correlation is significant at the 0.05 level (2-tailed)

Neither total bread nor wholegrain bread intake at baseline was associated with weight change during the 20-year follow-up. No differences in weight change across the sex-specific quartiles of bread intake were observed, and *p* for trend across the quartiles was non-significant for all regression models (Table 4). However, a higher white bread intake was associated with weight gain (Table 4). Sensitivity analyses including only those who reported consuming white bread at baseline (*n* = 533) showed that higher white bread intake was associated with weight gain in the model adjusted for baseline body weight (Model 1) and in the model adjusted for baseline body weight, sex, and energy intake (Model 2). However, this association did not remain significant in the fully adjusted model (Model 3; estimate: 0.019 kg/g white bread/day, 95% CI -0.003 to 0.041 kg/g white bread/day, *p* = 0.091) (Online Resource 2).

Sensitivity analyses adjusted for smoking and physical activity level at baseline did not change the strength, direction, or significance level of the associations between total bread, wholegrain bread, or white bread intake at baseline with weight change during follow-up (Online Resource 3).

### Wholegrain intake and weight change

Baseline wholegrain consumption was significantly and positively associated with wholegrain consumption at follow-up (Table 3). Overall, baseline wholegrain intake was inversely associated with weight gain (Table 4). However, weight change across quartiles of wholegrain consumption did not differ. Trend analyses across quartiles showed a significant association for wholegrain intake in regression model 1 and 2, but this was attenuated in the fully adjusted model (Table 4). Sensitivity analyses adjusted for smoking and physical activity level at baseline did not change the strength or direction of the association between wholegrain intake and weight change. However, the association no longer reached statistical significance (effect estimate: -0.01 kg/g whole grain/day, 95% CI: -0.02 to 0.00 kg/g whole grain/day, *p* = 0.140) (Online Resource 3).

**Table 4:** The associations of bread intake, wholegrain intake, and total plasma alkylresorcinol concentration with changes in body weight over 20 years in men and women participating in the Hordaland health studies

Absolute weight change (kg)				
Total cohort (*n* = 1764)				
	**Estimate (95% confidence interval)** ^a^		***p-*** **value** ^a^	
**Total bread**,** g/day**				
Model 1	0.000 (-0.005 to 0.004)		0.853	
Model 2	-0.002 (-0.007 to 0.005)		0.528	
Model 3	-0.002 (-0.007 to 0.003)		0.501	
**Wholegrain bread**,** g/day**				
Model 1	-0.003 (-0.007 to 0.001)		0.110	
Model 2	-0.003 (-0.007 to 0.001)		0.094	
Model 3	-0.003 (-0.007 to 0.001)		0.107	
**White bread**,** g/day**				
Model 1	0.021 (0.005 to 0.036)		**0.009**	
Model 2	0.020 (0.005 to 0.036)		**0.011**	
Model 3	0.017 (0.002 to 0.032)		**0.026**	
**Whole grains**,** g/day**				
Model 1	-0.014 (-0.026 to -0.001)		**0.029**	
Model 2	-0.018 (-0.031 to -0.004)		**0.009**	
Model 3	-0.013 (-0.026 to 0.000)		**0.047**	
**Total plasma alkylresorcinols**,** nmol/L**				
Model 1^b^	-0.005 (-0.008 to -0.003)		**< 0.01**	
Model 2^b^	-0.005 (-0.008 to -0.003)		**< 0.01**	
Model 3^b^	-0.004 (-0.007 to -0.002)		**< 0.01**	
**Quartiles of total bread intake**				***p*** **for trend**
Model 1				0.398
Q1 (108 ± 32 g bread/day, *n* = 442)	0.351 (-0.617 to 1.320)		0.477	
Q2 (158 ± 28 g bread/day, *n* = 442)	-0.310 (-1.279 to 0.658)		0.529	
Q3 (201 ± 37 g bread/day, *n* = 440)	-0.575 (-1.154 to 0.394)		0.245	
Q4 (274 ± 69 g bread/day, *n* = 440)	Ref.		-	
Model 2^c^				0.342
Q1 (108 ± 32 g bread/day, *n* = 442)	0.408 (-0.587 to 1.402)		0.422	
Q2 (158 ± 28 g bread/day, *n* = 442)	-0.271 (-1.252to 0.710)		0.589	
Q3 (201 ± 37 g bread/day, *n* = 440)	-0.546 (-1.522 to 0.430)		0.237	
Q4 (274 ± 69 g bread/day, *n* = 440)	Ref.		-	
Model 3^c^				0.334
Q1 (108 ± 32 g bread/day, *n* = 442)	0.401 (-0.559 to 1.361)		0.413	
Q2 (158 ± 28 g bread/day, *n* = 442)	-0.204 (-1.153 to 0.471)		0.673	
Q3 (201 ± 37 g bread/day, *n* = 440)	-0.471 (-1.415 to 0.471)		0.327	
Q4 (274 ± 69 g bread/day, *n* = 440)	Ref.		-	
**Quartiles of wholegrain intake**				
Model 1				**0.039**
Q1 (30 ± 8 g whole grain/day, *n* = 444)	0.910 (-0.56 to 1.877)		0.065	
Q2 (47 ± 6 g whole grain/day, *n* = 438)	0.534 (-0.435 to 1.504)		0.280	
Q3 (62 ± 8 g whole grain/day, *n* = 441)	0.050 (-0.919 to 1.018)		0.920	
Q4 (95 ± 24 g whole grain/day, *n* = 441)	Ref.		-	
Model 2^c^				**0.023**
Q1 (30 ± 8 g whole grain/day, *n* = 444)	1.101 (0.093 to 2.108)		**0.032**	
Q2 (47 ± 6 g whole grain/day, *n* = 438)	0.650 (-0.335 to 1.635)		0.196	
Q3 (62 ± 8 g whole grain/day, *n* = 441)	0.100 (-0.871 to 1.071)		0.840	
Q4 (95 ± 24 g whole grain/day, *n* = 441)	Ref.		-	
Model 3^c^				0.088
Q1 (30 ± 8 g whole grain/day, *n* = 444)	0.766 (-0.217 to 1.749)		0.126	
Q2 (47 ± 6 g whole grain/day, *n* = 438)	0.499 (-0.454 to 1.452)		0.305	
Q3 (62 ± 8 g whole grain/day, *n* = 441)	0.015 (-0.926 to 0.957)		0.974	
Q4 (95 ± 24 g whole grain/day, *n* = 441)	Ref.		-	

Model 1: adjusted for baseline body weight. Model 2: adjusted for baseline body weight, sex, and partial energy intake at baseline. Model 3: adjusted for baseline body weight, sex, partial energy intake at baseline, smoking and physical activity during follow-up, and educational attainment at baseline

^a^ Effect estimates, corresponding 95% confidence intervals and *p*-values are obtained by linear regression models

^b^ Not adjusted for sex

^c^ Additionally adjusted for baseline triglyceride concentration (mmol/L)

### Total plasma alkylresorcinols and weight change

Total wholegrain intake at baseline was significantly correlated with plasma AR concentrations (Spearman’s rho 0.27, *p* < 0.001). Total plasma AR concentrations were inversely associated with weight gain (Table 4). Sensitivity analyses adjusted for smoking and physical activity level at baseline did not change the strength, direction, or significance level of the association between plasma AR concentrations and weight change (Online Resource 3).

### Interpretation of estimates

For every slice (40 g) of refined wheat bread consumed daily, the β-coefficient of 0.017 kg/g white bread/day obtained from the regression analyses (Table 4) translates to an increase of 0.7 kg body weight during the 20-year follow-up. For every portion (30 g) of whole grains consumed daily, the β-coefficient of -0.013 kg/g whole grains/day obtained from the regression analyses translates to a decrease of 0.4 kg body weight during follow up. And lastly, for every 50 nmol/L increase in plasma AR concentration, the β-coefficient of -0.004 kg/nmol/L plasma AR obtained from the regression analyses translates to a decrease of 0.2 kg body weight during the 20-year follow-up period.

## Discussion

In this community-based cohort of Norwegian men and women, total bread intake was not associated with future changes in body weight, although analyses of breads differing in carbohydrate quality revealed that a higher consumption of refined white bread was associated with weight gain. Further, higher wholegrain consumption – but not wholegrain bread – was associated with less weight gained. In concordance, higher total plasma alkylresorcinol concentration was associated with less weight gained from middle to late adulthood.

Changes in dietary habits may occur throughout life, particularly in periods of significant life transitions [37]. These transitions may disrupt pre-existing habits and lead to changes in diet and dietary behaviours. Although the exposure variables in the current analyses were based on baseline dietary intake, they likely reflect dietary habits throughout the follow-up period. This assumption is supported by a recent paper based on the 1950-51 age group participating in both HUSK2 and HUSK3, indicating stable dietary patterns for most participants over the 20-year follow-up [38].

A modest weight gain was observed over the 20-year follow-up in the current study, and most participants had a stable weight from middle to late adulthood. Previous studies have found the rate of weight gain to be higher during young adulthood, levelling off, and stabilizing, or even declining, in older adulthood around the age of 60 [39, 40]. Unfortunately, we have no data on intermediate weight changes between the two study waves, and thus, we might have missed the peak weight in some of the participants. Together, these factors may partly explain the stable weight among most participants and the overall modest weight gain observed.

Although no association of overall bread intake with weight change from middle to late adulthood was present in the current cohort, the type of bread consumed seemed to play a role in weight regulation as consumption of white bread was associated with weight gain. This finding relates to results from a prospective cohort study assessing changes in bread consumption and adiposity where those with the highest increase in white bread consumption also gained most weight [1]. Another prospective cohort study found consumption of two or more portions of white bread daily at baseline to be associated with a higher risk of overweight and obesity during a 5-year follow-up [2]. Contrary, wholegrain bread consumption and dietary pattern including wholegrain bread have not been associated with weight gain nor with the risk of overweight or obesity [1, 2, 41]. This is in line with the findings from the current study where wholegrain bread consumption was not associated with weight gain. Thus, the health implications of bread consumption seem to be influenced by its carbohydrate quality.

In the current cohort, consumption of whole grains seemed to relate to improved body weight regulation. The similar associations of wholegrain consumption and plasma ARs may also underpin causation. Few prospective cohort studies have explored the association between plasma ARs and weight change, except for Kyrø et al. [42] who followed nearly 40.000 participants over 5 years, but measured ARs only in a subset of 516 subjects. As opposed to our finding, no association between plasma AR concentrations and self-reported weight was found, although higher plasma AR concentrations were associated with reduced waist circumference in women. Thus, the contrasting findings might reflect the comprehensive data collection in the current study where plasma AR levels were available from 1764 participants. Further, body weight was measured by trained personnel in the current study as opposed to self-reported weight used by Kyrø et al. [42]. And lastly, half of the participants with measured plasma ARs were diagnosed with colon cancer during follow-up in the study by Kyrø et al. [42], which might have influenced their weight and changed their way of living.

In line with findings from the current study, wholegrain consumption has been associated with lower weight measures in cross-sectional studies [6, 7, 43], and with less weight gained in prospective cohort studies [44–47]. However, inconsistencies exist and a lack of association between reported wholegrain intake and weight has been reported in observational studies [42], which might be attributable to how wholegrain foods are defined [48]. Additionally, the same beneficial effect of whole grains on weight is not always present in randomized controlled trials (RCTs) [49]. The lack of consistency between observational studies and RCTs has been explained by whole grain itself not necessarily being causally linked to weight regulation. Wholegrain consumption might be a marker for other health-related behaviours with weight-regulating properties, and the reported association in observational studies might be attributable to residual confounding. A higher wholegrain intake has been related to higher intakes of fruit and vegetables, dairy products, fish and shellfish [50], and with higher levels of physical activity and less smoking [51]. However, the lack of an effect of whole grains on weight in RCTs may also be due to the study durations being too short to capture weight changes, and participants may not be compliant with the interventions. Additionally, in a meta-analysis of RCTs summarising the effect of whole grains on body weight in healthy adults [49], 19 of the 26 included studies attempted to keep the body weight constant during the trial period. Further, there are several plausible metabolic and biological mechanisms that could explain an effect of whole grains on weight, including improved appetite regulation [52] which might be reflected by the potential influence whole grains have on gastrointestinal health [53], secretion of gastrointestinal hormones [54], and glucose metabolism [55].

To our knowledge, this is the largest prospective cohort study applying a quantitative dietary assessment method and a dietary biomarker in parallel to explore the association of bread and wholegrain intake with long-term weight changes from middle to late adulthood. While self-reported wholegrain intake might be prone to reporting bias, such as social desirability bias [56], ARs provide a more objective measurement of wholegrain intake. Thus, a major strength of the current study is the concurrent inclusion an FFQ and plasma ARs. Another strength is the well-characterised cohort, followed for 20 years, with clinical, biochemical, and dietary data available. Further, the prospective design allowed for exploration of dietary exposures in relation to subsequent weight changes during a period of life that is particularly important for healthy aging but also a period prone to weight changes and development of non-communicable diseases. Additionally, weight was measured according to standard procedures by trained personnel, which provides more accurate measures than self-reported weight [57].

A limitation of the current study is the infrequent collection of dietary data which may have resulted in less precise dietary exposure variables. As the dietary exposure was based on dietary intake at baseline, changes in food consumption and food preferences might have occurred during the follow-up period. Additionally, using an FFQ to estimate bread and wholegrain intake must be viewed as a crude method as detailed information on the specific bread types and the specific wholegrain content of different foods are limited. The FFQ used in this study does not provide detailed information on specific types of formulations of bread consumed. Bread products available on the market may have changed during the follow-up period in response to international trends, public health campaigns, and industry initiatives, potentially altering the nutritional composition of bread within the same category (e.g., wholegrain bread). Consequently, we cannot determine whether a wholegrain bread consumed at baseline was nutritionally equivalent to one consumed during the follow-up period. However, previous analyses have shown that the overall dietary pattern in the cohort remained relatively stable during the follow-up period between the second and third wave of the HUSK studies [38]. Furthermore, another limitation is that while we have data on total wholegrain consumption and intake of different bread types, we cannot determine the contribution of non-bread sources to the total wholegrain intake.

Compared with the general Norwegian population, the participants in the current study reported higher educational attainment [58], lower obesity rates, lower smoking rates, and higher levels of physical activity [59]. Thus, the presence of “healthy volunteer” selection bias may compromise the external validity of the findings. Additionally, a large proportion of the participants quit smoking during follow-up, maybe influenced by the 2004 smoking ban in Norway, prohibiting smoking in all indoor public spaces, including restaurants, bars, and workplaces [60]. This legislation, combined with national public health campaigns, likely motivated many smokers to quit. In addition, middle-aged individuals may also have been motivated by increasing health concerns, contributing to smoking cessation in this demographic. When comparing characteristics of participants who attended both HUSK2 and HUSK3 with those only attending HUSK2, previous analyses have demonstrated that completers and non-completers were comparable regarding body mass index and waist circumference, whereas a larger proportion of completers were non-smokers and had educational attainment > 12 years [61]. This suggest that while lifestyle and socioeconomic factors may have differed, baseline anthropometric measures were comparable. Additionally, we lacked information on the specific reasons for non-participation in HUSK3, and thus, we cannot rule out the possibility that attrition was related to unmeasured health factors or mortality. This may suggest the presence of some attrition bias, which requires the findings to be interpreted accordingly. Furthermore, although hormonal changes during menopause may impact weight regulation, all women in our study had transitioned to postmenopausal status by follow-up. Therefore, we did not include menopausal status as a covariate, but we acknowledge that menopause-related metabolic changes might have influenced weight trajectories.

Another limitation is the collection of non-fasting blood samples for analysing plasma AR as this is an acute biomarker with a relatively short half-life (approx. 5 h) [21], and thus, the time since last meal may have affected the analyses. However, despite the relatively short half-life, plasma ARs have been demonstrated to be useful biomarkers for assessing medium-to-long-term wholegrain wheat and rye intake in free-living populations [22, 23]. Additionally, as ARs are biomarkers for wholegrain wheat and rye, it will not capture consumption of other wholegrain sources such as oats and barley, which also are consumed in the Norwegian diet, though to a lesser extent than wheat. Another limitation of this study is that the FFQ captures food items rather than specific grain sources. While wheat is the predominant whole grain in Norway, the inability to distinguish between different wholegrain sources may introduce some uncertainty in our findings. Furthermore, as the FFQ reflects the food intake during past year and plasma AR concentrations reflect acute wholegrain wheat and rye intake, the associations between these two methods should thus be interpreted with caution. Lastly, the use of self-reported data on physical activity is a limitation as objective measures would have provided more accurate data.

To establish the relevance of bread and whole grains for weight regulation, longer-term studies with repeated anthropometric measures and dietary assessments, as well as blood samples to measure biomarkers of whole grains, are warranted. Additionally, measuring biomarkers reflecting whole grains from other cereals, such as avenanthramides and avenacosides for oat intake [16], would be beneficial. A key consideration in nutritional epidemiology is the method used for energy adjustment, as different approaches can influence the interpretation of findings [35]. In this study, the energy partition model was applied, which allows for the assessment of the exposure of interest independent of energy sources. We acknowledge that the partition model, while commonly applied in nutritional epidemiology, may have limitations in terms of causal interpretation, particularly if the assumptions of no residual confounding and equal effects of other energy-contributing components are not met. While alternative methods, such as the residual model or nutrient density model, could also be applied, the partition model aligns with our research objectives. The choice of energy adjustment method may influence the results, and this should be considered when interpreting our findings. Moreover, although we did not perform formal food substitution analyses, such as modelling the replacement of white bread with whole grain bread, this represents an important avenue for future research. Substitution modelling could provide additional insight into how changes in specific food choices, within an isocaloric framework, may influence long-term weight regulation.

## Conclusion

The current analyses from the Hordaland Health Study demonstrate the importance of investigating the carbohydrate quality of bread rather than total bread consumption, as different bread types may affect body weight differently. Although no association between wholegrain bread consumption and weight change was present, higher wholegrain consumption was associated with less weight gained. This finding was further reinforced by including alkylresorcinols as biomarkers of wholegrain wheat and rye intake in the analyses, confirming the association between wholegrain consumption and weight. Overall, our findings indicate that increasing wholegrain consumption in general, and reducing the intake of refined white bread, might promote weight regulation from middle to late adulthood.

## Electronic supplementary material

Below is the link to the electronic supplementary material.

Supplementary Material 1

Supplementary Material 2

Supplementary Material 3

Supplementary Material 4
