# Supplementary material for: Bread, wholegrain consumption and weight change from middle to late adulthood: a prospective cohort study
Source: Eur J Nutr. 2025 May 30;64(5):197. doi: 10.1007/s00394-025-03724-8 (PMC12125146; doi:10.1007/s00394-025-03724-8)
Supplement: Supplementary file 1 — Supplementary Material 1 [file 394_2025_3724_MOESM1_ESM.docx]

STROBE Statement—checklist of items that should be included in reports of observational studies

|  | Item No. | Recommendation | Page  No. | Relevant text from manuscript |  |
| --- | --- | --- | --- | --- | --- |
| **Title and abstract** | 1 | (*a*) Indicate the study’s design with a commonly used term in the title or the abstract | 1 | “… a prospective cohort study | |
|  |  | (*b*) Provide in the abstract an informative and balanced summary of what was done and what was found | 2-3 | See abstract. | |
| Introduction | | | |  | |
| Background/rationale | 2 | Explain the scientific background and rationale for the investigation being reported | 4-5 | “The role of bread consumption for weight regulation has been controversial, and there are few scientific studies investigating the association of bread consumption with longer-term weight changes from middle to late adulthood – which is a crucial period for healthy aging and a period associated with development of non-communicable diseases.” | |
| Objectives | 3 | State specific objectives, including any prespecified hypotheses | 5 | “The main objective of the current study was to investigate the association of overall bread intake at baseline with longer-term weight change by following a cohort of Norwegian men and women from middle to late adulthood, covering the 6^th^ and 7^th^ decade of life. Secondary objectives were to investigate the associations of bread quality, total whole-grain intake, and total plasma AR concentration with longer-term weight change.” | |
| Methods | | | |  | |
| Study design | 4 | Present key elements of study design early in the paper | 6 | “The Hordaland Health Studies are community-based health surveys conducted in Hordaland County, Western Norway. In the current study, data from the 1950-51 cohort in HUSK2 and HUSK3 were used to explore the associations of bread intake, whole-grain intake, and plasma AR concentrations with weight change between HUSK2 (baseline) and HUSK3 (follow-up).” | |
| Setting | 5 | Describe the setting, locations, and relevant dates, including periods of recruitment, exposure, follow-up, and data collection | 6-9 | “All residents of Hordaland County, Norway, born in 1925-27 and 1950-52 were invited to participate in the Hordaland Homocysteine Study in 1992-93. In 1997-99, the two age cohorts were reinvited to participate in the Hordaland Health Study (HUSK2). In 2018-20, those born in 1950-51 were again reinvited to take part in a follow-up health survey, HUSK3. In the current study, data from the 1950-51 cohort in HUSK2 and HUSK3 were used to explore the associations of bread intake, whole-grain intake, and plasma AR concentrations with weight change between HUSK2 (baseline) and HUSK3 (follow-up).” | |
| Participants | 6 | (*a*) *Cohort study*—Give the eligibility criteria, and the sources and methods of selection of participants. Describe methods of follow-up  *Case-control study*—Give the eligibility criteria, and the sources and methods of case ascertainment and control selection. Give the rationale for the choice of cases and controls  *Cross-sectional study*—Give the eligibility criteria, and the sources and methods of selection of participants | 7 | “In total, 2232 participants born in 1950-51 attended both HUSK2 and HUSK3. Participants who completed the food frequency questionnaire (FFQ) and provided measurements of body weight and plasma AR in HUSK2, and who provided data on body weight in HUSK3, were included (*n*=1799). Participants who reported a very low energy intake (<3000 kJ/day for women and <3300 kJ/day for men) or a very high energy intake (>15 000 kJ/day for women and >17 500 kJ/day for men), and participants with implausible total plasma AR values (>1200 nmol/L), were excluded. After removal of participants with missing exposure and outcome data (*n*=433), implausible energy intakes (*n*=32), and implausible plasma AR values (*n*=3), 1764 subjects were included in the statistical analyses.” Methods to follow-up: see section above. | |
|  |  | (*b*) *Cohort study*—For matched studies, give matching criteria and number of exposed and unexposed  *Case-control study*—For matched studies, give matching criteria and the number of controls per case |  |  | |
| Variables | 7 | Clearly define all outcomes, exposures, predictors, potential confounders, and effect modifiers. Give diagnostic criteria, if applicable | 11-12 | “The outcome assessed was change in body weight (kg) over a period of 20 years. Multivariate linear regression models were applied to examine the association between 1) total bread intake (g/day), 2) wholegrain bread intake (g/day), 3) white bread intake (g/day), 4) wholegrain intake (g/day), and 5) total plasma AR concentrations (nmol/L) at baseline with changes in body weight (kg) during follow-up. The exposure variables were expressed as continuous variables (g/day), and for total bread and wholegrain intake, both continuous and categorical (sex-specific quartiles) variables were applied. Sex-specific quartiles were used to get an equal distribution of males and females across the quartiles. The outcome variable, weight change (kg) during follow-up, was used as a continuous variable. In the regression analyses, Model 1 was adjusted for baseline body weight, Model 2 was adjusted for baseline body weight, sex, and partial energy intake at baseline, and Model 3 was adjusted for body weight, sex, partial energy intake, and education obtained at baseline, and changes in smoking habits and physical activity levels during follow-up.” | |
| Data sources/ measurement | 8* | For each variable of interest, give sources of data and details of methods of assessment (measurement). Describe comparability of assessment methods if there is more than one group | 7-10 | “In both surveys, body weight was measured in light clothing without shoes to the nearest 0.5 kg on a calibrated digital scale.” See the method section for description of all variables included in the analyses. | |
| Bias | 9 | Describe any efforts to address potential sources of bias | 12 | “In the regression analyses, Model 1 was adjusted for baseline body weight, Model 2 was adjusted for baseline body weight, sex, and partial energy intake at baseline, and Model 3 was adjusted for body weight, sex, partial energy intake, and education obtained at baseline, and changes in smoking habits and physical activity levels during follow-up.” | |
| Study size | 10 | Explain how the study size was arrived at |  | Not applicable as we used all participants available that fulfilled the inclusion criteria. | |

Continued on next page

| Quantitative variables | 11 | Explain how quantitative variables were handled in the analyses. If applicable, describe which groupings were chosen and why | 11 | “The exposure variables were expressed as continuous variables (g/day), and for total bread and wholegrain intake, both continuous and categorical (sex-specific quartiles) variables were applied.” |
| --- | --- | --- | --- | --- |
| Statistical methods | 12 | (*a*) Describe all statistical methods, including those used to control for confounding | 12 | “In the regression analyses, Model 1 was adjusted for baseline body weight, Model 2 was adjusted for baseline body weight, sex, and partial energy intake at baseline, and Model 3 was adjusted for body weight, sex, partial energy intake, and education obtained at baseline, and changes in smoking habits and physical activity levels during follow-up.” |
|  |  | (*b*) Describe any methods used to examine subgroups and interactions | 12-13 | “…, sensitivity analyses including only those who reported consuming white bread the past year (*n* = 533) were conducted …” |
|  |  | (*c*) Explain how missing data were addressed | 12 | “Missing data (*n* = 2 for smoking, *n* = 11 for education, and *n* = 84 for physical activity level) were accounted for by applying imputation methods.” |
|  |  | (*d*) *Cohort study*—If applicable, explain how loss to follow-up was addressed  *Case-control study*—If applicable, explain how matching of cases and controls was addressed  *Cross-sectional study*—If applicable, describe analytical methods taking account of sampling strategy |  | Not applicable. |
|  |  | (*e*) Describe any sensitivity analyses | 12-13 | “As the overall baseline intake of white bread was low, sensitivity analyses including only those who reported consuming white bread in the past year (*n* = 533) were conducted. Furthermore, as the main analyses incorporated changes in smoking habits and physical activity levels during follow-up as covariates, sensitivity analyses using smoking status and physical activity level at baseline were conducted. Sensitivity analyses were also conducted where those who started smoking during follow-up (*n* = 6) were excluded from the statistical analyses as the small size of this group could introduce some instability in the statistical model. The “new smokers” were excluded rather than combined with another group as smoking habits have a large impact on body weight, and thus, combining categories might introduce bias. These analyses can be viewed in **Online Resource 1**.” |
| Results | | | | |
| Participants | 13* | (a) Report numbers of individuals at each stage of study—eg numbers potentially eligible, examined for eligibility, confirmed eligible, included in the study, completing follow-up, and analysed | 14 | “The study included 1764 participants of whom 1024 (58%) women and 740 (42%) men.” |
|  |  | (b) Give reasons for non-participation at each stage | 7 | In total, 2232 participants born in 1950-51 attended both HUSK2 and HUSK3. Participants who completed the food frequency questionnaire (FFQ) and provided measurements of body weight and plasma AR in HUSK2, and who provided data on body weight in HUSK3, were included (*n*=1799). Participants who reported a very low energy intake (<3000 kJ/day for women and <3300 kJ/day for men) or a very high energy intake (>15 000 kJ/day for women and >17 500 kJ/day for men), and participants with implausible total plasma AR values (>1200 nmol/L), were excluded. After removal of participants with missing exposure and outcome data (*n*=433), implausible energy intakes (*n*=32), and implausible plasma AR values (*n*=3), 1764 subjects were included in the statistical analyses. |
|  |  | (c) Consider use of a flow diagram |  | See Figure 1. |
| Descriptive data | 14* | (a) Give characteristics of study participants (eg demographic, clinical, social) and information on exposures and potential confounders | 14-16 | “Several distinctions were present when comparing participants across quartiles of total bread intake including differences in heigh, BMI, total cholesterol, and triglycerides (Table 1).” Table 1 and Table 2 contains participant characteristics. |
|  |  | (b) Indicate number of participants with missing data for each variable of interest |  | Not applicable as we imputed missing values. |
|  |  | (c) *Cohort study*—Summarise follow-up time (eg, average and total amount) | 14 | “The median duration of follow-up was 20 years, ranging from 19 to 22 years.” |
| Outcome data | 15* | *Cohort study*—Report numbers of outcome events or summary measures over time |  | *Not applicable.* |
|  |  | *Case-control study—*Report numbers in each exposure category, or summary measures of exposure |  |  |
|  |  | *Cross-sectional study—*Report numbers of outcome events or summary measures |  |  |
| Main results | 16 | (*a*) Give unadjusted estimates and, if applicable, confounder-adjusted estimates and their precision (eg, 95% confidence interval). Make clear which confounders were adjusted for and why they were included | 18-20 | See Table 4. |
|  |  | (*b*) Report category boundaries when continuous variables were categorized | 14-20 | Information found in Table 1, Table 2, and Table 4. |
|  |  | (*c*) If relevant, consider translating estimates of relative risk into absolute risk for a meaningful time period |  | Not applicable. |

Continued on next page

| Other analyses | 17 | Report other analyses done—eg analyses of subgroups and interactions, and sensitivity analyses | 14-21 | | “Sensitivity analyses including only those who reported consuming white bread at baseline (*n* = 533) showed that higher white bread intake was associated with weight gain in the model adjusted for baseline body weight (Model 1) and in the model adjusted for baseline body weight, sex, and energy intake (Model 2). However, this association did not remain significant in the fully adjusted model (Model 3; estimate: 0.019 kg/g white bread/day, 95% CI -0.003 to 0.041 kg/g white bread/day, *p* = 0.091) (**Online Resource 2**).” |
| --- | --- | --- | --- | --- | --- |
| Discussion | | | | | |
| Key results | 18 | Summarise key results with reference to study objectives | | 22 | “In this community-based cohort of Norwegian men and women, total bread intake was not associated with future changes in body weight, although analyses of breads differing in carbohydrate quality revealed that a higher consumption of refined white bread was associated with weight gain. Further, higher wholegrain consumption – but not wholegrain bread – was associated with less weight gained. In concordance, higher total plasma alkylresorcinol concentration was associated with less weight gained from middle to late adulthood.” |
| Limitations | 19 | Discuss limitations of the study, taking into account sources of potential bias or imprecision. Discuss both direction and magnitude of any potential bias | | 25-27 | “A limitation of the current study is the infrequent collection of dietary data which may have resulted in less precise dietary exposure variables...” |
| Interpretation | 20 | Give a cautious overall interpretation of results considering objectives, limitations, multiplicity of analyses, results from similar studies, and other relevant evidence | | 22-27 | “Though no association of overall bread intake with weight change from middle to late adulthood was present in the current cohort, the type of bread consumed seemed to play a role in weight regulation as consumption of white bread was positively associated with weight gain. This finding can be related to results from a prospective cohort study assessing changes in bread consumption and adiposity where those with the highest increase in white bread consumption also gained most weight (1).” |
| Generalisability | 21 | Discuss the generalisability (external validity) of the study results | | 25 | “Compared with the general Norwegian population, the participants in the current study reported higher educational attainment (58)lower obesity rates, lower smoking rates, and higher levels of physical activity (59). Thus, the presence of “healthy volunteer” selection bias in the current study may compromise the external validity of the findings.” |
| Other information | |  | | | |
| Funding | 22 | Give the source of funding and the role of the funders for the present study and, if applicable, for the original study on which the present article is based | | 29 | “Open access funding was provided by the University of Bergen. The current study received funding from the Research Council of Norway, Formas Research Council of Sweden, the Federal Ministry of Education and Research (Germany) under the umbrella of the European Joint programming Initiative "A healthy diet for a health life" (JPI-HDHL) and of the ERA-NET Cofund HDHL INTIMIC (GA N 727565 of the EU Horizon 2020 Research and Innovation Programme). The funders were not involved in the design of the study, nor in the collection, entry, analysis, interpretation of data, or writing of the article.” |

*Give information separately for cases and controls in case-control studies and, if applicable, for exposed and unexposed groups in cohort and cross-sectional studies.

**Note:** An Explanation and Elaboration article discusses each checklist item and gives methodological background and published examples of transparent reporting. The STROBE checklist is best used in conjunction with this article (freely available on the Web sites of PLoS Medicine at http://www.plosmedicine.org/, Annals of Internal Medicine at http://www.annals.org/, and Epidemiology at http://www.epidem.com/). Information on the STROBE Initiative is available at www.strobe-statement.org.
