# Supplementary material for: Bread, wholegrain consumption and weight change from middle to late adulthood: a prospective cohort study
Source: Eur J Nutr. 2025 May 30;64(5):197. doi: 10.1007/s00394-025-03724-8 (PMC12125146; doi:10.1007/s00394-025-03724-8)
Supplement: Supplementary file 2 — Supplementary Material 2 [file 394_2025_3724_MOESM2_ESM.pdf]

Bread, wholegrain consumption and weight change from middle to late adulthood: a prospective cohort study.
European Journal of Nutrition.
Revheim I, Sabir Z, Dierkes J, Buyken EA, Landberg R, Alten, Spielau U, Rosendahl-Riise H

Corresponding author
Hanne Rosendahl-Riise
University of Bergen, Department of Clinical Medicine, Bergen, Norway

The associatio of bread intake, wholegrair intake, anc total plasn alkylresorc concentra with chang in body we over 20 yea in men anc women participati in the Hordaland Health Studies.

Table s1. The associations of bread intake, wholegrain intake, and total plasma alkylresorcinol concentration with changes in body weight over 20 years in men and women participating in the Hordaland Health Studies.

	Absolute weight change (kg) Total cohort (n = 1758)	
	**Estimate (95% confidence interval)[1]**	***p* value[1]**
**Total bread, g/day**		
Model 1	0.000 (-0.005 to 0.004)	0.853
Model 2	-0.002 (-0.007 to 0.004)	0.562
Model 3	-0.002 (-0.007 to 0.003)	0.531
**Wholegrain bread, g/day**		
Model 1	-0.003 (-0.007 to 0.001)	0.110
Model 2	-0.003 (-0.007 to 0.000)	0.083
Model 3	-0.003 (-0.007 to 0.001)	0.094
**White bread, g/day**		
Model 1	0.020 (0.005 to 0.036)	**0.011**
Model 2	0.020 (0.004 to 0.036)	**0.013**
Model 3	0.017 (0.001 to 0.032)	**0.032**
**Whole grains, g/day**		
Model 1	-0.014 (-0.026 to -0.001)	**0.031**
Model 2	-0.018 (-0.031 to -0.004)	**0.009**

Bread, wholegrain consumption and weight change from middle to late adulthood: a prospective cohort study.
European Journal of Nutrition.
Revheim I, Sabir Z, Dierkes J, Buyken EA, Landberg R, Alten, Spielau U, Rosendahl-Riise H

Corresponding author
Hanne Rosendahl-Riise
University of Bergen, Department of Clinical Medicine, Bergen, Norway

Model 3	-0.013 (-0.026 to 0.000)	**0.046**
**Total plasma alkylresorcinols, nmol/L**		
Model 1[b]	-0.005 (-0.008 to -0.003)	**<0.01**
Model 2[b]	-0.005 (-0.008 to -0.003)	**<0.01**
Model 3[b]	-0.004 (-0.007 to -0.002)	**<0.01**
**Quartiles of total bread intake**		
Model 1		
Q1 (108 ± 32 g bread/day, *n* = 440)	0.336 (-0.634 to 1.306)	0.497
Q2 (158 ± 28 g bread/day, *n* = 441)	-0.298 (-1.266 to 0.671)	0.547
Q3 (201 ± 37 g bread/day, *n* = 439)	-0.516 (-1.485 to 0.454)	0.297
Q4 (275 ± 69 g bread/day, *n* = 438)	Ref.	-
Model 2[c]		
Q1 (108 ± 32 g bread/day, *n* = 440)	0.399 (-0.596 to 1.394)	0.432
Q2 (158 ± 28 g bread/day, *n* = 441)	-0.253 (-1.235 to 0.728)	0.613
Q3 (201 ± 37 g bread/day, *n* = 439)	-0.483 (-1.460 to 0.493)	0.332
Q4 (275 ± 69 g bread/day, *n* = 438)	Ref.	-
Model 3[c]		
Q1 (108 ± 32 g bread/day, *n* = 440)	0.395 (-0.565 to 1.356)	0.420
Q2 (158 ± 28 g bread/day, *n* = 441)	-0.190 (-1.139to 0.758)	0.694
Q3 (201 ± 37 g bread/day, *n* = 439)	-0.412 (-1.355 to 0.531)	0.327
Q4 (275 ± 69 g bread/day, *n* = 438)	Ref.	-
**Quartiles of wholegrain intake**		
Model 1		
Q1 (30 ± 8 g whole grain/day, *n* = 442)	0.876 (-0.091 to 1.877)	0.076
Q2 (47 ± 6 g whole grain/day, *n* = 436)	0.576 (-0.393 to 1.545)	0.244
Q3 (62 ± 8 g whole grain/day, *n* = 439)	0.028 (-0.940 to 0.995)	0.956
Q4 (95 ± 24 g whole grain/day, *n* = 441)	Ref.	-
Model 2[c]		
Q1 (30 ± 8 g whole grain/day, *n* = 442)	1.075 (0.068 to 2.082)	**0.036**
Q2 (47 ± 6 g whole grain/day, *n* = 436)	0.698 (-0.286 to 1.683)	0.164
Q3 (62 ± 8 g whole grain/day, *n* = 439)	0.081 (-0.890to 1.052)	0.870
Q4 (95 ± 24 g whole grain/day, *n* = 441)	Ref.	-
Model 3[c]		
Q1 (30 ± 8 g whole grain/day, *n* = 442)	0.739 (-0.242 to 1.721)	0.140
Q2 (47 ± 6 g whole grain/day, *n* = 436)	0.548 (-0.404 to 1.500)	0.259
Q3 (62 ± 8 g whole grain/day, *n* = 439)	-0.005 (-0.945 to 0.936)	0.992

Bread, wholegrain consumption and weight change from middle to late adulthood: a prospective cohort study. European Journal of Nutrition.
Revheim I, Sabir Z, Dierkes J, Buyken EA, Landberg R, Alten, Spielau U, Rosendahl-Riise H

Corresponding author
Hanne Rosendahl-Riise
University of Bergen, Department of Clinical Medicine, Bergen, Norway

Q4 (95 ± 24 g whole grain/day, *n* = 441)	Ref.	-

Model 1: adjusted for baseline body weight
Model 2: adjusted for baseline body weight, sex,and baseline energy intake
Model 3: adjusted for baseline body weight, sex, baseline energy intake, changes in smoking and physical acitivity levels during follow-up, and education obtained at baseline.
[1] Effect estimates, corresponding 95% confidence intervals and *p*-values are obtained by linear regression models.
[2] Additionally adjusted for baseline triglycerid concentration (mmol/L).
Those starting smoking during the follow-up duration (n = 6) were excluded from the analyses.
