# Supplementary material for: Bread, wholegrain consumption and weight change from middle to late adulthood: a prospective cohort study
Source: Eur J Nutr. 2025 May 30;64(5):197. doi: 10.1007/s00394-025-03724-8 (PMC12125146; doi:10.1007/s00394-025-03724-8)
Supplement: Supplementary file 3 — Supplementary Material 3 [file 394_2025_3724_MOESM3_ESM.pdf]

Bread, whole-grain consumption and weight change from middle to late adulthood: a prospective cohort study. European Journal of Nutrition.

Revheim I, Sabir Z, Dierkes J, Buyken EA, Landberg R, Alten, Spielau U, Rosendahl-Riise H

Corresponding author
Hanne Rosendahl-Riise
University of Bergen, Department of Clinical Medicine, Bergen, Norway

Table s2. Association of refined white bread consumption and weight change during the 20-year follow-up of Norwegian men and women reporting white bread consumption at baseline in the Hordaland Health Studies

	**Absolute weight change (kg)**			
	**Cohort (*n* = 533)**			
	**Estimate (95% CI)[1]**	***p* value[1]**	**R^2 [1]**	***p* value (model)[1]**
**White bread (g/day)**				
Model 1	0.025 (0.003 to 0.047)	**0.023**	0.011	0.018
Model 2	0.025 (0.003 to 0.047)	**0.029**	0.009	0.068
Model 3	0.019 (-0.003 to 0.041)	0.091	0.059	<0.001
Model 4	0.019 (-0.003 to 0.042)	0.085	0.032	0.001

Model 1: adjusted for baseline body weight

Model 2: adjusted for baseline body weight, sex and partial energy intake at baseline
Model 3: adjusted for baseline body weight, sex, partial energy intake at baseline, smoking and physical acitivity habits during follow-up, and education obtained at baseline
Model 4: Adjusted for covariates obtained at baseline: body weight, sex, partial energy intake, education, smoking and physical acitivity

[1] Effect estimates, corresponding 95% confidence intervals (CI) and *p*-values are obtained from linear regression models.
