# Supplementary material for: Bread, wholegrain consumption and weight change from middle to late adulthood: a prospective cohort study
Source: Eur J Nutr. 2025 May 30;64(5):197. doi: 10.1007/s00394-025-03724-8 (PMC12125146; doi:10.1007/s00394-025-03724-8)
Supplement: Supplementary file 4 — Supplementary Material 4 [file 394_2025_3724_MOESM4_ESM.pdf]

Bread, whole-grain consumption and weight change from middle to late adulthood: a prospective cohort study.
European Journal of Nutrition.
Revheim I, Sabir Z, Dierkes J, Buyken EA, Landberg R, Alten, Spielau U, Rosendahl-Riise H

Corresponding author
Hanne Rosendahl-Riise
University of Bergen, Department of Clinical Medicine, Bergen, Norway

Table s3. The associations of bread intake, whole-grain intake, and plasma alkylresorcinol concentration with weight change during 20-year follow-up in Norwegian men and women participating in the Hordaland Health Studies.

Absolute weight change (kg)				
	**Total cohort (*n* = 1764)**			
	**Estimate (95% CI)[1]**	***p* value[1]**	**R^2 [1]**	***p* value (model)[1]**
**Bread intake, g/day**	-0.001 (-0.006 to 0.004)	0.608	0.060	<0.001
**Whole-grain bread intake, g/day**	-0.002 (-0.006 to 0.002)	0.275	0.061	<0.001
**White bread intake, g/day**	0.016 (0.001 to 0.031)	**0.042**	0.059	<0.001
**Whole-grain intake, g/day**	-0.010 (-0.023 to 0.003)	0.140	0.058	<0.001
**Total plasma AR concentration, nmol/L**	-0.004 (-0.006 to -0.001)	**0.007**	0.064	<0.001
**Quartiles of total bread intake**			0.057	<0.001
Q1 (108 ± 32 g bread/day, *n* = 442)	0.338 (-0.638 to 1.313)	0.497		
Q2 (158 ± 28 g bread/day, *n* = 442)	-0.214 (-1.176 to 0.749)	0.664		
Q3 (201 ± 37 g bread/day, *n* = 440)	-0.531 (-1.488 to 0.426I	0.277		
Q4 (274 ± 69 g bread/day, *n* = 440)	ref.			
**Quartiles of whole-grain intake**			0.057	<0.001
Q1 (30 ± 8 g whole grains/day, *n* = 444)	0.487 (-0.512 to 1.487)	0.339		
Q2 (47 ± 6 g whole grains/day, *n* = 438)	0.376 (-0.593 to 1.344)	0.447		
Q3 (62 ± 8 g whole grains/day, *n* = 441)	-0.195 (-1.150 to 0.761)	0.690		
Q4 (95 ± 24 g whole grains/day, *n* = 441)	ref.			

The linear regression model is adjusted for the covariates body weight, sex, energy intake, current smoking (yes/no), physical acitivity level, and education, all obtained at baseline.
[1] Effect estimates, corresponding 95% confidence intervals (CI) and *p*-values are obtained from linear regression models.

Bread, whole-grain consumption and weight change from middle to late adulthood: a prospective cohort study.
European Journal of Nutrition.
Revheim I, Sabir Z, Dierkes J, Buyken EA, Landberg R, Alten, Spielau U, Rosendahl-Riise H

Corresponding author
Hanne Rosendahl-Riise
University of Bergen, Department of Clinical Medicine, Bergen, Norway
